# Blood from ‘junk’: the LTR chimeric transcript *Pu.2* promotes erythropoiesis

**DOI:** 10.1186/1759-8753-5-15

**Published:** 2014-05-09

**Authors:** Kyle R Upton, Geoffrey J Faulkner

**Affiliations:** 1 Mater Research Institute – University of Queensland, TRI Building, 4102 Brisbane, QLD, Australia; 2 School of Biomedical Sciences, University of Queensland, 4072 Brisbane, QLD, Australia

**Keywords:** LTR retrotransposon, Transposable element, *ORR1A0*, KLF1, KLF3, Erythroid, *Pu.1*, *Pu.2*

## Abstract

Transposable elements (TEs) are a prominent feature of most eukaryotic genomes. Despite rapidly accumulating evidence for the role of TE-driven insertional mutagenesis and structural variation in genome evolution, few clear examples of individual TEs impacting biology via perturbed gene regulation are available. A recent report describes the discovery of an alternative promoter for the murine erythroid transcription factor *Pu.1*. This promoter is located in an *ORR1A0* long terminal repeat (LTR) retrotransposon intronic to *Pu.1* and is regulated by the Krüppel-like factors KLF1 and KLF3. Expression of the resultant chimeric transcript, called *Pu.2*, spontaneously induces erythroid differentiation *in vitro*. These experiments illustrate how transcription factor binding sites spread by retrotransposition have the potential to impact networks encoding key biological processes in the host genome.

## Background

Transposable elements (TEs) have in the past been maligned as ‘junk’ [1], ‘selfish’, and ‘parasitic’ [2,3]. These descriptions are likely apt for many if not most sequences derived from TEs. However, a more complete view is that the majority of DNA generated by TE activity evolves neutrally under selection, while a small yet important minority of TE-derived sequences continues to drive genome evolution and innovation. The potential importance of TEs to the host is suggested by their near ubiquitous presence in eukaryotes, often accounting for half or more of genome sequence content [4-6] and, more convincingly, their provision of regulatory or otherwise functional genetic elements [7]. In primates, for example, most order-specific regulatory sequences are derived from TEs [8]. Many of these regions are dynamically regulated during development [9,10] and incorporate internal binding sites for suppressor and activator complexes [11].

As a TE proliferates in its host genome, the number of loci subject to regulation by DNA binding proteins specific to that TE, such as transcription factors, also increases. During evolution, TEs have often contributed transcription factor binding sites to promoter, enhancer, and boundary elements [9-14]. In some cases, entire developmental pathways have been rewired as a result of TE mobilization [15-17]. Novel TE insertions can result in alternative splicing [18], exonization [19,20], altered mRNA translational efficiency [21,22], as well as the provision of distal enhancers [23]. Thus, an abundance of TE-derived sequences, including ready-made promoters, enhancers, and other regulatory units, points to a major role for TEs in shaping the regulatory landscape of the eukaryotic genome (see [24] for a recent review).

### Discovery and characterization of *Pu.2*, an LTR-driven chimeric mRNA

Despite extensive evidence for gene regulatory and structural innovation produced by TEs, examples of phenotypic change due to this variation are comparatively limited in mammals. TE-derived alternative promoters, which generate a chimeric mRNA with an adjacent gene, are arguably one of the more straightforward scenarios to link a TE with a functional product, particularly when that gene encodes a protein of known function. In recent work, Mak *et al.*[25] report the discovery and functional characterization of a long terminal repeat (LTR) promoted chimeric mRNA of *Pu.1*, regulated in turn by Krüppel-like factors 1 (KLF1) and 3 (KLF3). PU.1, KLF1, and KLF3 are transcription factors active during hematopoiesis, where PU.1 favors myeloid differentiation and KLF1 and KLF3 drive erythroid maturation [26,27]. In wild-type mice, KLF1 and KLF3 recognize similar sequence motifs in an antagonistic manner. KLF1 generally acts as a transcriptional activator [27], while KLF3 recruits a repressive complex including epigenetic modifiers [28].

**Figure 1:**
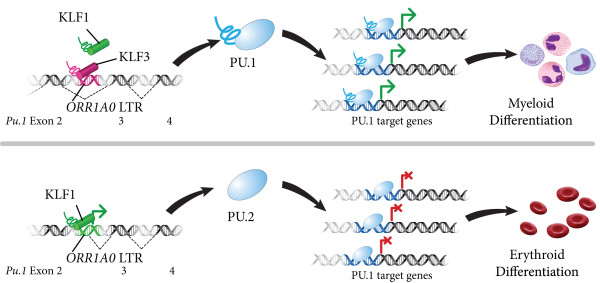
**A potential role for *Pu.2 *in erythroid differentiation, as described by Mak *et al.*** PU.1 is a master regulator of myeloid differentiation (top). KLF1 and KLF3, respectively, activate and repress an alternative promoter included in an *ORR1A0* LTR located in the second intron of *Pu.1*. KLF3-mediated suppression of this LTR is the norm in wild-type fetal liver cells, permitting canonical PU.1 expression and myeloid differentiation. In the absence of KLF3, Mak *et al.* found the *ORR1A0* LTR produced a chimeric mRNA with *Pu.1* that, in turn, encoded a truncated protein isoform called PU.2 (bottom) lacking the N-terminal activation domain of PU.1. PU.2 retained its DNA binding capacity, but did not interact with other transcription factors, leading to a putative role as a dominant negative isoform of PU.1 promoting erythroid maturation.

Analyzing a microarray screen of *Klf3*^-/-^ knockout mice, Mak *et al.* first identified major de-repression of *Pu.1* that, oddly, excluded the initial two exons of the gene. To resolve this discrepancy, the authors performed 5′ RACE primed from the third exon of *Pu.1*. The results of this approach indicated an alternative promoter in an *ORR1A0* LTR [29] located in the second intron of *Pu.1* (Figure 1). Subsequent qRT-PCR assays confirmed that the *ORR1A0*-*Pu.1* chimeric transcript, named *Pu.2*, was upregulated in *Klf3*^-/-^ fetal liver tissue, while KLF1 and KLF3 were found to bind sequence motifs contained in *ORR1A0*, based on electrophoretic mobility shift assay (EMSA) and luciferase reporter experiments. Taken together, these data suggest opposing roles for KLF1 and KLF3 in regulating *Pu.2* expression.

Intriguingly, *Pu.2* was predicted to encode an N-terminal truncated isoform of PU.1 that retained a DNA binding ETS domain (Figure 1). By EMSA, Mak *et al.* demonstrated that PU.2 could bind to a predicted PU.1 target site. Moreover, PU.2 was found to counter the activity of PU.1 in a dose-dependent manner, and promoted spontaneous erythroid differentiation in human K562 cells. These assays clearly demonstrated, *in vitro*, an erythropoietic function for *Pu.2*. Extending their results to an *in vivo* setting, the authors detected *Pu.2* mRNA and protein in wild-type fetal liver, with overexpression observed in *Klf3*^-/-^ animals, and also found that *in vitro* KLF1 overexpression activated PU.2. These experiments provide evidence for PU.2 function in murine erythroid differentiation, albeit heavily repressed by KLF3 *in vivo*.

Finally, via RNA-seq and additional qRT-PCR, Mak *et al.* identified several other *ORR1A0* alternative promoters differentially regulated by KLF3 and producing chimeric transcripts with adjacent protein-coding genes. This important, though preliminary, observation suggested that *ORR1A0* may play a broader role in regulating erythroid differentiation beyond the highlighted example of *Pu.2*, and provides insight into the co-evolution of TE subfamilies, transcription factors, and core biological processes, as discussed elsewhere [30]. One reasonable conclusion from this work is that the amplification of the *ORR1A0* LTR family in rodents generated a ready-made network of genetic material subject to control by KLF1 and KLF3, and capable of changing how erythroid maturation was regulated during development. Notably, the *ORR1A0* LTR family is rodent-specific and almost certainly incapable of further mobilization [29]. As such, *Pu.2* is not found in human cells, though it remains to be determined whether other TEs present in the human *Pu.1* locus generate mRNAs functionally analogous to mouse *Pu.2*. Future experiments involving genome-wide chromatin immunoprecipitation sequencing (ChIP-seq) to elucidate KLF bound sites *in vivo* may reveal human TE families dynamically regulated hematopoiesis, in the *Pu.1* locus and elsewhere.

## Conclusions

Among a host of alternative promoters derived from mammalian TEs and driving protein-coding and non-coding gene expression [9,31-33], we consider three reports as landmark examples of TE insertions having a clear functional impact upon biology: an epigenetically regulated LTR upstream of the *Agouti* gene in rodents [34], an LTR alternative promoter for the colony stimulating factor 1 receptor (CSF1R) proto-oncogene in lymphoma [35], and the convergent evolution of multiple LTRs to act as promoters for the neuronal apoptosis inhibitory protein (NAIP) gene [36]. In each case, an LTR produces a chimeric mRNA with the adjacent protein-coding gene. The discovery by Mak *et al.* that the LTR-initiated *Pu.2* transcript can promote erythroid maturation in the absence of KLF3 is a valuable addition to this literature, and will likely increase future attention to the role of TEs in regulating various developmental processes, including hematopoiesis.

## Abbreviations

Brca2: Breast cancer 2, early onset; ChIP-seq: Chromatin immunoprecipitation sequencing; CSF1R: Colony stimulating factor 1 receptor; EMSA: Electrophoretic mobility shift assay; Klf: Krüppel-like factor; LTR: Long terminal repeat; NAIP: Neuronal apoptosis inhibitory protein; qRT-PCR: quantitative real time PCR; RACE: Rapid amplification of cDNA ends; RNA-seq: RNA sequencing; TE: Transposable element.

## Competing interests

The authors declare that they have no competing interests.

## Authors’ contributions

GJF wrote the manuscript. KRU provided edits and prepared Figure 1. Both authors read and approved the final manuscript.
